# Reprogramming human cells to naïve pluripotency: how close are we?

**DOI:** 10.1016/j.gde.2017.06.009

**Published:** 2017-06-29

**Authors:** Lawrence E Bates, José CR Silva

**Affiliations:** Wellcome Trust Medical Research Council Cambridge Stem Cell, Institute and Department of Biochemistry, University of Cambridge, Tennis Court Road, Cambridge CB2 1QR, UK

## Abstract

Pluripotent stem cells (PSCs) have the potential to revolutionise biomedical science; however, while it is simple to reproducibly obtain comparable, stable cell lines in mouse, those produced from human material typically show significant variability both within and between cell lines. This is likely due to differences in the cell identity of conventional mouse and human PSCs. It is hoped that recently identified conditions to reprogram human cells to a naïve-like state will produce better PSCs resulting in reproducible experimental outcomes and more consistent differentiation protocols. In this review we discuss the latest literature on the discovery of human naïve-like stem cells and examine how similar they are to both mouse naïve cells and the preimplantation human epiblast.

## Introduction

Studies in mouse embryonic stem cells (mESCs) over many years have led to a detailed understanding of this cell state. While mouse cells are typically grown in a state of naïve pluripotency, equivalent to the naïve epiblast of the preimplantation blastocyst [1], human cells are cultured in primed pluripotency conditions. These are more similar to the postimplantation epiblast where cells become primed for differentiation [2]. Consequently, there are significant difficulties in directly applying our knowledge from mouse ESCs to human systems.

There have been several attempts to generate human naïve pluripotent stem cells (nPSCs) over recent years. Most often when putative human naïve cells are generated *in vitro* they are analysed using criteria that are known to distinguish mouse naïve cells from primed cells. Such criteria include responses to extrinsic and intrinsic signalling pathways, the biophysical, biochemical and metabolic status of the cells, and the overall epigenetic and transcriptomic cell identity. However, recent advances in our understanding of the human embryo also allow direct comparisons to the naïve compartment *in vivo*. Recently, cells exhibiting human naïve epiblast molecular features have been described [3••,4••,5••]. Over the course of this review we shall examine how closely these match the state of both mouse naïve ESCs and what is known of the human blastocyst.

## Transcriptional identity

**Figure 1:**
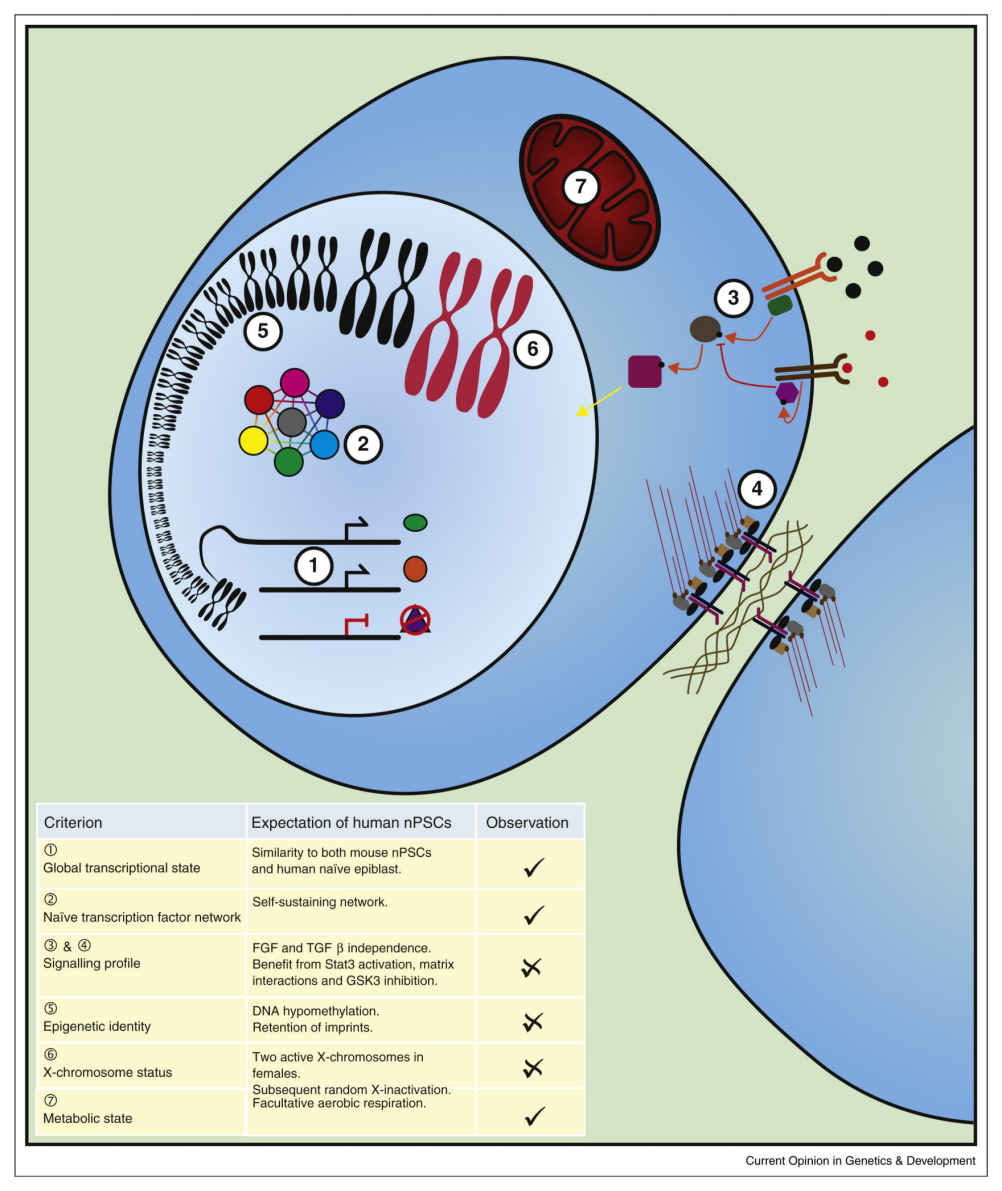
Expected molecular signatures of human naïve pluripotent stem cells. A large number of processes control, and are influenced by, any cell state. Some of the factors that are particularly considered in this review are: (**1**) the transcriptional state of the cell. Functional components such as Oct4 and Nanog and marker genes such as Rex1 have been identified from mouse naïve cells and the human preimplantation epiblast, building a fingerprint of gene expression that should be present in naïve cells. (**2**) A core transcription factor network. The naïve state in mouse has a self-sustaining network of transcription factors with many positive feedback loops to promote the maintenance of pluripotency. Notably, while many of these transcription factors are still expressed in primed cells, the network conformation is different, with factors binding to different enhancer elements and thus interacting in different ways. By exploring these interconnections, it is possible to test whether putative human naïve cells share the same connectivity and hence whether the network exists in a naïve configuration. (**3**,**4**) Environmental signals are key to maintaining cell states. In mouse, the naïve state can be maintained *in vitro* with LIF which activates downstream JAK/STAT signalling, an inhibitor of MEK/ERK signalling downstream of the FGF receptor, and an inhibitor of β-catenin degradation. The current human naïve culture conditions extend this with addition of a PKC inhibitor [4••], or BRAF, SRC and ROCK inhibitors [3••]. In addition to the response to ligands, cells interact physically with their neighbours and the extracellular matrix. Strong adherens junctions between cells provide the familiar dome-shaped morphology of naïve ESC colonies, and the ability to sense neighbours appears to be important for cell survival. (**5**) The epigenetic fingerprint of cells can be analysed in a similar manner to the transcriptional identity to build up a global picture of the cell state. Enhancer and promoter usage result in modification of histones and differential methylation of DNA. These profiles can be compared between cells. Additionally, the naïve state has additional epigenetic properties, such as global DNA hypomethylation and retention of imprinting marks which should be found in human naïve cells. (**6**) A key feature of the naïve state in female mouse cells is the presence of two active X-chromosomes. While the exact connection between naïve identity and X-chromosome status is still elusive, this is considered a hallmark of the naïve identity. While aspects of X-chromosome regulation differ between mouse and human, recent embryo work suggests that the human preimplantation epiblast shares this feature with mouse. (**7**) Many other elements of the cell are controlled by the cell state. One example is the switch between aerobic and anaerobic respiration. The naïve compartment of the embryo is considered to be facultatively aerobic, displaying relatively mature mitochondria, whereas other early embryonic cell states rely on anaerobic glycolysis for most of their energy requirements. While the cause of this switch is unknown, it is likely to be the result of integrating many other state-specific signals.

The transcriptional identity of a cell is often considered to be a readout of the cell’s state (Figure 1, part 1). However, it is clear that the transcriptional state of cells is plastic, and a range of genes fluctuate in response to intracellular and extracellular conditions. Historically, it has not been practical to get a large number of high quality human embryos, necessary to obtain replicates of transcriptomic data with sufficient depth. Also, such embryos may be compromised as they have to be cultured *in vitro* in order to generate blastocyst-like embryos. Importantly, recent advances in RNA sequencing, particularly protocols for small cell numbers and even single cell sequencing, have made the analysis of these embryos possible.

Using such techniques, Yan *et al.* [6], and more recently Blakeley *et al.* [7], obtained single-cell transcript data from human embryos including late blastocyst stage embryos. Yan *et al.* observed four distinct cell types by unsupervised clustering which appear to represent two trophectoderm populations as well as extra-embryonic endoderm and epiblast cells based on their expression of known marker genes, as expected in a mature blastocyst. However, both studies identified only a handful of epiblast cells, giving a fairly small sample size for further analysis.

Comparing the human naïve induced pluripotent stem cells (hniPSCs) of Takashima *et al.*, the embryo derived human naïve ESCs (hnESCs) from Guo *et al.* [5], the embryonic naïve epiblast cells from Yan and from Blakeley, as well as mouse nESCs and conventional primed human ESCs, it is clear that the hniPSCs and embryo-derived hnESCs cluster closely together [5]. This indicates that they share a transcriptional identity. Notably, the human naïve-like cell lines cluster closer to mouse ESCs than to human blastocyst cells along a principle component of variation. It is possible that this separation is the result of generic adaptation of cells to *in vitro* culture. Interestingly, established human primed lines are separated from primed epiblast outgrowths along this same axis.

Theunissen *et al.* took a different approach to comparing their datasets to published human embryo data. They identified genes that are expressed in specific embryonic stages in the dataset of Yan *et al.* They then looked for the proportion of these genes that were differentially expressed between their hniPSCs and conventional primed hESCs. While genes specifically expressed in embryonic epiblast were enriched in the hniPSCs, so were genes specific to the morula and all other cell types of the late blastocyst [8•]. This is surprising, since unsupervised clustering of all single cells in the study by Yan *et al.* [6] indicated that these are well defined states, with epiblast cells segregating well from other cells of the blastocyst. As stated by Theunissen *et al.* [8•], this may indicate that their hniPSCs are in an earlier embryonic state, perhaps before full segregation of the inner cell mass. By analysing the expression of transposable elements, they found striking similarities between their cells and morula blastomeres. Interestingly, recent work from Petropoulos *et al.* [9•] suggests that the segregation of lineages occurs relatively late in human embryos, with co-expression of lineage-specific genes throughout the morula. The three lineages only begin to segregate on the onset of blastocyst formation.

## Transcription factor network

At the core of the naïve cell identity in mouse ESCs is a highly interconnected transcription factor network which shows remarkable redundancy [10,11] (Figure 1, part 2). Whereas the complete transcriptome may not be identical between cell lines and across passages, these factors are always expressed in naïve cells. They are also functionally conserved across a range of vertebrates in their ability to drive induction of naïve pluripotent stem cells [12–14]. Additionally, many of these factors are specific for nPSCs compared to primed post-implantation epiblast derived stem cells (EpiSCs) [2] making these a good subset of genes to use as markers of a naïve state. Takashima, Guo and Theunissen all investigated a panel of these genes and found that most were upregulated in their putative hniPSCs and hnESCs relative to primed cells [3••,4••,5••]. Neither *ESRRB* nor *KLF2* were upregulated in any of these naïve lines; however, this may be due to differences between primate and rodent and the redundant use of paralogue genes such as Klf4 [1,4••,^7^]. It is interesting, however, that both Takashima and Theunissen were able to efficiently induce a naïve-like state through exogenous expression of *KLF2* alongside *NANOG*. Takashima elegantly demonstrated that the behaviour of the transcription factor network in his hniPSCs closely corresponded to that of mouse ESCs with a knockout and rescue strategy. Mouse ESCs can support the single loss of either *Esrrb* or *Tfcp2l1* due to redundancies in the network [15,16], but it was expected that double knockout would lead to network collapse and subsequent differentiation [4••]. Accordingly, shRNA depletion of *TFCP2L1* in hniPSCs resulted in greatly reduced colony formation, indicating that most cells had stopped self-renewing. Application of exogenous *ESRRB* during this knockdown was able to rescue self-renewal. Together this provides strong evidence that an interactive transcription network highly similar to that in mouse is active in these cells.

## Exogenous ligands and intracellular signalling landscape

A broad array of signalling pathways interact to maintain or destabilise the naïve state in mouse ESCs (Figure 1, part 3). These cells are able to self-renew in the absence of external signals providing that certain pro-differentiation pathways are blocked, namely the MEK/ERK MAPK signalling axis and the GSK3β pathway which would otherwise destabilise the network in part through its role in the degradation of β-catenin [17]. However, these conditions are not optimal and extrinsic signals that enhance pluripotency and survivability are often added to the culture. Most common, is the LIFR/GP130 ligand LIF which activates the JAK/STAT pathway [18,19]. This is typically included with inhibitors of GSK3β and MEK to give chemically defined 2i LIF medium. This enhances the efficiency of induced naïve pluripotency [17,20] and maintains mouse naïve PSCs in a state closely resembling the naïve epiblast of the pre-implantation embryo [1,17,20].

Given the importance of LIF and downstream JAK/STAT signalling in reprogramming and maintenance of mouse naïve PSCs [21,22] and its ability to induce human cells with some naïve-like properties [23,24], it was surprising that Theunissen *et al.* [3••] found hLIF to be dispensable for culture of their hniPSCs. Indeed their microarray data (publically accessible on the GEO database, accession GSE59435) indicates that LIFR expression is fourfold lower in naïve cells than in their parental primed cells. In addition, Takashima *et al.* [4••] show that LIFR is expressed at a far lower level in hniPSCs than in mouse ESCs. While LIF signalling is a key feature of the mouse blastocyst and is important for survival in diapause [25], it is also known to be important in the process of implantation in both mouse and humans [26,27]. Further work will be required to test whether JAK/STAT signalling is indeed an important component of the human naïve state.

In mouse, Fgf2 and Activin A are both used to support the undifferentiated growth of primed EpiSCs [2,28,29], while Fgf signalling leads to differentiation of naïve ESCs and Activin signalling is dispensable [17,29–31]. While the current human naïve cells can be maintained in the absence of FGF and Activin [3••,4••], Theunissen *et al.* identified increased differentiation on their double inhibition [3••]. Notably in mouse, following activation by LIF stimulation, JAKs activate PI3K and the AKT signalling pathway [32]. This appears to have a role in ESC self-renewal, with cells treated with a PI3K or PDK1 inhibitor showing reduced proliferation and increased multi-lineage differentiation [33,34]. The PI3K/AKT pathway is known to be activated downstream of both FGF and Activin A/TGFβ signalling in various contexts [29,35,36], but most interestingly FGF2-induced AKT activation has been demonstrated in conventional primed human ESCs, where it is proposed to boost cell survival [37]; in this manner, FGF/Activin could be beneficial to the human naïve-like state in the absence of sufficient LIF signalling to induce the AKT signalling cascade.

Given the poor survival of hniPSCs Theunissen *et al.* [3••] found it necessary to maintain these with ROCK inhibitor. While ROCK inhibitor was found not to be strictly required by Takashima et al. [4••], it was used in combination with their t2iL+Gö in feeder-free culture and embryo derivation [4••,5••]. In mouse, poor survival on single-cell passaging is a more common trait in primed cells [2], which can be similarly rescued with application of ROCK inhibitor [38]. The precise manner in which ROCKi contributes to the enhancement of self-renewal is not clear. It appears that following single-cell dissociation, loss of focal adhesions between cells leads to activation of RHO/ROCK signalling. This results in enhanced actinomyosin contractility which induces apoptosis [39,40]. The improved proliferation observed while culturing the hniPSCs in the presence of ROCKi may suggest that they are not responding appropriately to cell-cell and cell-substrate contacts (Figure 1, part 4). In mouse, a switch from E-cadherin expression in naïve ESCs to N-cadherin in primed EpiSCs has been observed [41,42]. Examination of the microarray data from Theunissen *et al.* reveals a decrease in N-Cadherin on transition to the naïve state; however, both N-cadherin and E-cadherin are expressed in primed human ESCs, and there is no further increase in E-cadherin in the naïve cells [8•]. It could be interesting to examine other cell contact-sensing pathways such as YAP/TAZ signalling to see if they are compromised. Interestingly, it has been suggested that overexpression of YAP promotes the reprogramming of human primed cells to a naïve-like identity [43].

## Epigenetic fingerprint

Another distinctive feature of most cell states, and particularly the naïve state, is the epigenetic landscape (Figure 1, part 5). In mouse ESCs cultured in 2i LIF, there is a remarkable genome-wide reduction in DNA methylation which is also observed *in vivo* [44–46]. It has been unclear until recently whether this should also be expected in humans [47,48]. However, recent work suggests that DNA methylation shows the same trend in human as in mouse [49]. In line with this, Theunissen, Guo and Takashima all identified a decrease in global DNA methylation [4••,5••,8•], on the order of that observed in human embryos [49]. Beyond this global decrease, however, there are signs that DNA methylation may not be properly regulated in these cells. Imprinted loci are specifically marked by methylation on one of the parental chromosomes. Stable imprints are retained throughout development and can still be found in differentiated cells. Importantly, they are observed in primed human ESCs. On conversion to naïve conditions, however, many of these marks are lost [50•], which is an issue as this is linked to poor differentiation *in vitro* and shows links to developmental disorders and tumourogenisis [51].

Another epigenetic property of mouse naïve ESCs is the absence of a silent X-chromosome in females resulting in the presence of two active X-chromosomes (Figure 1, part 6). On fertilisation, the paternal X-chromosome is specifically inactivated [52] and is only reactivated in the naïve epiblast at the blastocyst stage [52–55]. Shortly after implantation of the embryo a random X-chromosome is inactivated [56]. There has been some debate over the state in human blastocysts [57,58], but recent evidence shows that they do have two active X-chromosomes [9•]. In the early human female blastocyst, cells exhibit twice the amount of X-linked gene expression compared to counterpart male embryo cells [9•]. However, despite maintaining biallelic expression as the blastocyst develops further, transcription of X-linked genes is downregulated, a phenomenon termed ‘dampening’ of the X-chromosome [9•].

The status of the X-chromosome in female primed cells has also been somewhat contentious [59–61]. Primed cells have undergone X-inactivation, and there is no reactivation when reprogramming human somatic cells to primed iPSCs [61]. However, over prolonged culture of primed pluripotent cells, a phenomenon of erosion of X-inactivation can be observed [60–62]. It appears that expression of Xist, the master regulator of X-inactivation, becomes epigenetically silenced resulting in the subsequent activation of genes on the formerly inactive chromosome. This makes the presence of two active X chromosomes in primed human ESCs an epigenetic abnormality rather than a molecular feature of biological significance.

Recently, it was shown that in the reprogramming of human primed cells to a naïve-like state the silent X-chromosome reactivates [8•,63•]. Despite exhibiting biallelic expression, it was found that X-linked gene expression was not twice that of the cells of origin which contained a silent X chromosome [63•]. Instead it resembled the ‘dampening’ phenomenon observed *in vivo* in very late human blastocysts [8•,9•,63•]. Upon differentiation there was inactivation of the X chromosome. However, this was non-random and therefore not reflective of the process that occurs during development.

Together, these studies indicate that there are epigenetic differences between current human nPSCs, their *in vivo* counterpart, and mouse ESCs.

## Conclusion

By the majority of measures, the most up to date culture systems have produced human pluripotent cells with similarities to both mouse naïve ESCs and to the human preimplantation epiblast. Nonetheless, there are still significant discrepancies. The signalling pathways active in these cells and the transcription factor network they support, appear to be very similar to, yet far less stable than, their equivalents in mouse ESCs. It is currently not possible to say whether the reduced stability of the human naïve state is due to interspecies differences, suboptimal culture conditions, or the possibility that we have not yet isolated bona fide human nPSCs.

Evidence from Takashima and from Guo show that their naïve cells have undergone metabolic reprogramming, showing a significant level of mitochondrial respiration (Figure 1, part 7) [4••,5••]. This is typically associated with mature blastocysts, with cells before and after this stage relying more heavily on anaerobic respiration and displaying less mature mitochondria [64,65]. Additionally, naïve cells show increased glycolytic metabolism, inhibition of which appears to reduce their proliferation, demonstrating that the high level of metabolic activity is important to the maintenance of these cells [66]. On the other hand, some transcriptional data suggest that the culture systems may favour a less mature embryonic state. Finally, several issues such as reduced viability in single cell passaging and genomic instability could indicate suboptimal conditions for cell growth.

Interestingly, recent papers have identified novel hPSCs with broader chimerism and differentiation potential than naïve or primed cells. These respectively demonstrate the ability to form interspecies chimaeras and the ability to differentiate into both embryonic and extraembryonic lineages *in vivo* [67,68]. The latter cells express naïve marker genes but show also an expression signature that is not similar to any embryonic cell type. Further characterisation of the novel human PSCs is now needed to ascertain their full properties and molecular signatures.

The next major hurdles in establishing hnPSCs as the standard for *in vitro* studies will require demonstrating superior differentiation potential and reliability compared to conventional human ES cultures, and methods to simplify the generation and culture of these cells. By identifying cell surface markers specific to hnPSCs, Collier *et al.* [69] present a step forward in facilitating the establishment of naïve cultures, as well as potentially allowing the study of reprogramming intermediates to dissect this interesting transition.

While the conditions for differentiation protocols may need to be optimised for these new cells, it will be important to learn whether the promises of more homogeneous, less cell-line dependent differentiation from a naïve starting population can be delivered. If so, then this cell state could take over to become the accepted standard starting point for drug discovery models, *in vitro* developmental studies, and possibly advances in cell therapies.
